# Barriers to male involvement in contraceptive uptake and reproductive health services: a qualitative study of men and women’s perceptions in two rural districts in Uganda

**DOI:** 10.1186/1742-4755-11-21

**Published:** 2014-03-05

**Authors:** Allen Kabagenyi, Larissa Jennings, Alice Reid, Gorette Nalwadda, James Ntozi, Lynn Atuyambe

**Affiliations:** 1 Department of Population Studies, College of Business and Management Sciences, Makerere University, P. O. Box 7062, Kampala, Uganda; 2 Center for Population and Statistics, College of Business and Management Sciences, Makerere University, P. O. Box 7062, Kampala, Uganda; 3 Department of International Health, Johns Hopkins Bloomberg School of Public Health, Social and Behavioral Interventions, 615 N. Wolfe Street, E5038, Baltimore, MD 21205, USA; 4 Department of Geography, University of Cambridge, Downing Pl, Cambridge CB2 3EN, UK; 5 Department of Nursing, College of Health Sciences, Makerere University, P. O. Box 7062, Kampala, Uganda; 6 Department of Community and Behavioral Sciences, School of Public Health, College of Health Sciences, Makerere University, P. O. Box 7062, Kampala, Kampala, Uganda

**Keywords:** Male involvement, Contraception, Family planning, Reproductive health, Qualitative research, Uganda

## Abstract

**Background:**

Spousal communication can improve family planning use and continuation. Yet, in countries with high fertility rates and unmet need, men have often been regarded as unsupportive of their partner’s use of family planning methods. This study examines men and women’s perceptions regarding obstacles to men’s support and uptake of modern contraceptives.

**Methods:**

A qualitative study using 18 focus group discussions (FGDs) with purposively selected men aged 15–54 and women aged 15–49 as well as eight key informant interviews (KIIs) with government and community leaders was conducted in 2012 in Bugiri and Mpigi Districts, Uganda. Open-ended question guides were used to explore men and women’s perceptions regarding barriers to men’s involvement in reproductive health. All FGDs and KIIs were recorded, translated, and transcribed verbatim. Transcripts were coded and analyzed thematically using ATLAS.ti.

**Results:**

Five themes were identified as rationale for men’s limited involvement: (i) perceived side effects of female contraceptive methods which disrupt sexual activity, (ii) limited choices of available male contraceptives, including fear and concerns relating to vasectomy, (iii) perceptions that reproductive health was a woman’s domain due to gender norms and traditional family planning communication geared towards women, (iv) preference for large family sizes which are uninhibited by prolonged birth spacing; and (v) concerns that women’s use of contraceptives will lead to extramarital sexual relations. In general, knowledge of effective contraceptive methods was high. However, lack of time and overall limited awareness regarding the specific role of men in reproductive health was also thought to deter men’s meaningful involvement in issues related to fertility regulation.

**Conclusion:**

Decision-making on contraceptive use is the shared responsibility of men and women. Effective development and implementation of male-involvement family planning initiatives should address barriers to men’s supportive participation in reproductive health, including addressing men's negative beliefs regarding contraceptive services.

## Introduction

Research suggests that male involvement can increase uptake and continuation of family planning methods by improving spousal communication through pathways of increased knowledge or decreased male opposition [1-5]. Yet, despite growing evidence on the benefits of engaging men in reproductive health decision-making, fertility rates and unmet need for family planning remain high in many sub-Saharan African countries. While there are many influential factors, low contraceptive prevalence has been attributed in part to men’s opposition to or non-involvement in family planning [6-8]. Male engagement has historically been depicted as obstructive by impeding women’s decision-making on use of family planning, or non-existent among male partners who are absent altogether due to lack of interest in matters related to reproductive health [9]. However, at the same time, men dominate decision-making regarding family size and their partner’s use of contraceptive methods in many traditionally patriarchal settings [10,11]. Women point to their male partner’s resistance to family planning as a significant barrier to uptake and continuation, resulting in decisions to use contraceptive methods covertly or not at all [12,13]. Fear of spousal retaliation due to disagreements about whether to use contraception has also been shown to be a significant barrier among women [14]. This seemingly contradictory role among men of being both key decision-makers regarding fertility desires and remaining detached from reproductive health issues has posed considerable challenges in African contexts to involve men to address low contraceptive prevalence rates [15,16].

While family planning services have traditionally targeted women, there is growing recognition that reproductive health is the joint responsibility of men and women. Given that men often have significant influences on a couple’s contraceptive use [17], pilot programs to engage men have focused on increasing knowledge, enhancing spousal communication, and de-stigmatizing use of family planning methods. Renewed interests in involving men stem not only from women’s reproductive health needs, but also to address men’s own sexual health concerns, as well as efforts to achieve the Millennium Development Goals (MDGs) for reduction of maternal mortality and HIV transmission. Use of modern contraception and family planning services is integral in the prevention of unwanted pregnancy, reduction of unsafe abortions, and promotion of childbirth spacing to lower maternal and child mortality risks in developing countries [18-21]. Family planning also promotes gender equity and greater educational and economic opportunities for women [6]. Several small-scale initiatives aiming to include men in reproductive health programs have had positive experiences [22], but in-depth understanding of the rationale for men’s low participation has been underexplored. This is urgently needed in the development and scale-up of evidence-based male-involvement family planning interventions.

Using qualitative research methods, this study examines men and women’s perceptions regarding obstacles to men’s support and uptake of modern contraceptives. Qualitative research methods facilitate in-depth understanding of the socio-cultural underpinnings of health behaviors and health motivations. Yet, there is a dearth of literature on factors which hinder men’s involvement in reproductive health from the perspective of men themselves. The few studies available have largely been conducted in non-African countries with dissimilar cultural settings and varying efforts to integrate men’s views with those of women and local stakeholders [23-26]. Studies conducted in African contexts have found that limited knowledge about family planning is a key determinant of men’s negative perception of and lack of engagement in family planning [27] as well as gender norms regarding men’s roles [28-30]. Some studies suggest also that spousal communication is low even in cases where men approve of family planning [27,31]. Despite these initial findings, less is known regarding the full range of men’s perspectives towards male and female contraceptive use. To address this gap, this study examines gendered views regarding factors limiting men’s involvement, as evidenced by partner communication, approval, support, or utilization of family planning methods and its implications for future research and intervention design.

## Methods

### Study design

A cross-sectional qualitative study was conducted using focus group discussions (FGDs) with women aged 15 – 49 and men aged 15 – 54 as well as 8 key informant interviews with government and community leaders in Bugiri and Mpigi Districts, Uganda.

### Setting

Uganda has an estimated population of 34 million people and is one of the youngest populations in the world [32]. Over 56% of the population is under 18 years of age, and the country has a growth rate of 3.2% per annum [32]. Recent national data has highlighted current reproductive health challenges. More than half of all pregnancies are unintended and roughly a quarter of maternal deaths are due to complications from unsafe abortions [33]. Uganda has one of the highest fertility rates in the region at an average of 6.7 children per woman, although the majority of Ugandan women would prefer fewer children [34]. Within the country, the contraceptive prevalence rates range from 19 to 30% [33,34], and the unmet need for family planning, referring to the estimate of women who desire to delay or prevent pregnancy but are not using contraception, is 36% [35]. Women and men’s access to modern contraceptive methods is also limited in some settings [34]. Traditional gender norms within Uganda elevate men as primary decision-makers in women’s use of family planning methods, although spousal communication and utilization of reproductive health services among men remains low [34,36].

### Participant selection

All study participants were purposively selected. Comparable to standard reproductive age categories, men aged 15–54 and women aged 15–49 in current married or non-married partnerships who were living in Bugiri and Mpigi Districts at the time of the study and willing to participate were eligible to join. Bugiri and Mpigi Districts were selected to provide a range of contexts for contraceptive uptake. District health officers, members of village health teams, council leaders, and representatives from local women and men’s groups were recruited for key informant interviews. Participants were selected from both rural and urban settings within each of the two districts with the help of local field guides and community leaders.

### Data collection

Data were collected in July and August 2012. Open-ended, semi-structured question guides were used to explore perceptions regarding barriers to men’s involvement in reproductive health. Discussions and interviews were conducted in the local languages, Lusoga and Luganda, in Bugiri and Mpigi Districts, respectively, until saturation was reached and no new findings emerged during study team debriefings. Interviews and focus group discussions were held in audibly private areas. Data were gathered by two trained research assistants with experience conducting qualitative research. The lead author of the study supervised all data collection to ensure quality control and assisted in taking notes. All study participants were encouraged to openly discuss their opinions. No personal information in the form of names or other identifying data was obtained.

### Data analysis

All discussions and interviews were recorded and transcribed verbatim in Lusoga and Luganda. After validating the transcription, the typed narratives were then translated into English and verified for accuracy. Analysis of the data was conducted by the primary author and included several iterative steps. Using thematic content analyses, the transcripts were reviewed several times, and a set of codes were developed to describe groups of words, or categories, with similar meanings. Transcripts were then coded and managed using ATLAS.ti (Version 7). The grouped categories were refined and used to generate themes emerging from the data. Direct quotations from men, women, and community key informants are presented in italics to highlight key findings.

### Ethical consideration

This study received ethics approval by the Makerere University School of Statistics and Planning Ethics Committee and the Uganda National Council of Science & Technology. Local leaders in each of the two districts were also invited to review and approve the study. Prior to data collection, informed consent was obtained for all potential study participants. Only the research team had access to the study data.

## Results

A total of 18 FGDs, eight male and ten female groups, as well as 8 KIIs were conducted. This represented a total of 154 individuals, 70 men and 84 women, respectively, who participated in the study. The average duration of FGDs and informant interviews was 90 minutes. The majority of participants were married or cohabiting and had completed secondary education. Five key themes were identified as reasons for men’s limited involvement in reproductive health. These included: side effects of female contraceptive methods, dissatisfaction with male contraceptive choices, perceptions that family planning was a woman’s domain, large family size preferences, and fear of partner sexual promiscuity.

### Side effects of female contraceptive methods

A commonly reported disincentive among men to support their partner’s use of contraceptive methods related to perceived side effects which were blamed for reducing sexual pleasure and increasing women’s risks of infertility and illness. Men reported being frustrated by several observed side effects, most notably irregular and prolonged bleeding, as well as vaginal dryness, and decreases in sex drive or libido. Excessive bleeding in particular was seen as having detrimental effects on marriages as long periods of blood loss reportedly led to women’s general fatigue and dampened their interests in sexual intercourse. Bleeding was also attributed to limiting the number of opportunities for men to have sex with their partner. This was seen as a precursor and motivation for developing extramarital sexual relations.

“Some of [the] women lose their sexual appetite, and no longer want to be with a man and others bleed for all the three months. Sometimes this causes problems in the marriages. When you are with your wife, the feeling is as though you do not have a wife. One ends up looking for sex outside their marriage”. Male FGD Participant, Mpigi

“They over bleed throughout. So as men, when you find her over bleeding, you choose to go out for other women. So, we want you to give us some advice because even going for vasectomy has a problem”. Male FGD Participant, Bugiri

While women were perceived as having the physical burden of contraceptive side effects, men considered themselves to be indirectly affected by side effects, resulting in requests by several men that their spouses discontinue contraceptive use altogether. In addition to women’s reportedly decreased interest in sex due to contraceptive side effects, excessive bleeding was also associated with vaginal odors.

“When you have a wife in the house and she is bleeding, the man does not have an opportunity to enjoy sexual intercourses with her. But now, just tell me if you have treated her and she gets fine. Tell me what will happen. Would you accept her to go back for family planning methods? You cannot allow her to go back, so that is the problem”. Male FGD participant Bugiri

“Men are against family planning, saying that women who go for family planning services never reach their sexual orgasm, bleed for so long, and also stink a lot. Such men refuse their spouses from going for family planning services so they end up producing more children”. Female Key Informant, Bugiri

Other perceived side effects which motivated men to oppose women’s use of contraception related to concerns in delayed return or permanent loss of fertility, as well as fears that short-term methods, such as birth control pills or injections, could lead to congenital abnormalities.

“These pills affect the fetus while in the uterus, sometimes women give births to children without some body parts, for instance a child may have no arms and sometimes some other body parts. We are told that pills are the cause of those abnormalities”. Male FGD Participant, Bugiri

Men equated the side effects of family planning methods also with having adverse economic effects on the household. Women’s reports of dizziness, nausea, and tiredness were thought to affect women’s ability to endure the physical demands of agricultural labor, resulting in a reduction in household productivity. Direct or indirect losses in revenue were further exacerbated by additional medical care costs to treat women’s discomfort. While side effects such as pain, mood changes, and breast tenderness were less commonly mentioned, such symptoms may have also contributed to men’s unsupportive involvement.

“If you have a wife that swallows pills, these pills have some side effects on those women like dizziness. The others get nausea. Yet, for us in our area, we are farmers. When they [women] go to the garden, they return late… without any work done [and] complaining of sickness. If your wife has no side effects in using family planning pills then it is okay. But the problem is that women ask for money from us [men] because they are sick and need treatment for the side effects. Some [women] lose energy while others lose their libido and get tired of sleeping with men”. Male FGD Participant, Mpigi

“You find that the money you spend treating your wife because of the problems she got after using family planning methods is more than the money you would have used treating a live child”. Male FGD Participant, Bugiri

### Dissatisfaction with male contraceptive choices

A second theme related to perceptions that while men were dissatisfied with the perceived side effects of female contraceptives, the two available male contraceptive methods were equally unappealing, namely male condoms and vasectomy, the surgical removal of sperm ducts. Limited access to a more diverse set of male-led methods was cited as additional motivation for men’s disapproval of family planning. The permanence and irreversibility of vasectomy was noted, in particular, as inacceptable among men and consistent with losing one’s masculinity.

“In every 100 men, you can only find one man or even none practicing family planning. You might not find even one man going to the health center for family planning services because we have only one method of contraception from the health facility which is vasectomy which lasts until death. However, women have many short term methods such as injections, pills, coils, and many others”. Male FGD Participant, Mpigi

“We men have no family planning pills, but we are afraid of what we have for family planning like vasectomy which is equal to castration. I am really afraid of such a method. That is the problem that we men have. Otherwise, we would participate in family planning. ..We only have two methods. Whereby, if you don’t use a condom, then you have to go for vasectomy”. Male FGD Participant, Mpigi

Given the expansion of modern contraceptive methods designed for women, this raises the possibility of unmet need for family planning among men who in some cases expressed desires to limit child birth due to the financial burden of raising large families. Preferences for a male-version of birth control pills were proposed as a potentially convenient male-led method to limit family size. Participants suggested that such short-term technologies for men would increase men’s interest in and uptake of family planning services.

“If we had pills for family planning, as men, that would help us. Maybe we would be involved. For us who want to plan our families, we would be involved [in the] use of contraceptives and family planning. I have paid a lot of school fees and felt the heavy burden. I would have stopped delivering long time ago but because we do not have family planning pills for men, we only have one method of vasectomy”. Male FGD Participant, Mpigi

“If we had something like a string that we would tie somewhere to space children and when it reaches a time when you want more children you would untie, then we would directly get involved in family planning to avoid giving birth to children who roam the streets”. Male FGD Participant, Mpigi

Women echoed similar sentiments relating to the lack of diversity among male contraception methods. There were also views that older men considered male condoms as designed predominantly for unmarried and younger individuals, and thus not well tailored for older sexually active men. Use of male condoms was also associated with distrust among couples.

“Condoms, especially youths, use them to avoid impregnating ladies and also to avoid HIV/AIDS. Other men especially the adults like in their 50s do not want family planning and have a negative attitude towards it. Men regularly say that use of condoms is a waste of time and can even ruin their homes”. Female Key Informant, Mpigi

### Perceptions of family planning as a woman’s domain

Social norms as well as health system factors were also identified as stymieing men’s participation in reproductive health services. Men and women highlighted gender norms which assigned the role of childbearing and child-rearing to women. Matters relating to fertility and birth planning were also considered to be within this domain. Engaging men in communication regarding family planning was perceived by some as inappropriate and distractive. Given the social expectations for men to earn income for their families, use of men’s limited time and mental preoccupation to discuss family planning was considered unduly burdensome.

“No, men do not have time and do not want to know anything related to contraceptives. They usually say that such things are for women, since they are responsible for carrying the pregnancy. For them [men], their [men’s] responsibility is to look for money”. Male Key Informant, Mpigi

“Sometimes men have no time. They are busy looking for money. So it’s useless to involve them in such issues of family planning and contraceptive uses since their minds are thinking about money. Usually, it is us women who produce children. Therefore, we carry the burden [of] being pregnant. Therefore, we do not see why we should involve men in such issues”. Female Key Informant, Mpigi

At the same time, men’s lack of involvement was blamed on family planning services, including awareness-raising campaigns, which have traditionally targeted women. This was thought to further define family planning as a woman’s domain, including initiating partner discussions and managing contraception. In cases where male involvement was perceived appropriate, lack of knowledge about how to be involved was often cited by participants as another deterrent for men.

“Men are not involved family planning and promotion of contraceptive use because they do not know. It is common for women to be sensitized because they go for antenatal [care], but the men do not go for antenatal [care]. There are only few who go there. At our village, there are no sensitizations targeting men. It is only a few women who educate their men, and they can’t explain to them very well”. Male FGD Participant, Mpigi

“Sensitize all men about family planning and also train them about their roles when it comes to family planning. By doing so, they will come to know the importance of family planning in a home, as most have no idea at all”. Female Key Informant, Mpigi

### Family size preferences

The absence of men’s support of women’s contraceptive use was additionally linked with patrilineal traditions that highly value children and encourage large family sizes. Numerous children were described as a sign of wealth and financial security.

“Men think children are a source of security especially if they are boys, usually if one builds on the right, another left, then people will fear to attack you because you already have security. Again when asking for votes like me if you have many children definitely those are many votes already and you will win”. Male Key Informant, Bugiri

“Most men do not like the issue of family planning because it reduces the size of their families. Men also have negative attitudes towards birth control because they say if you reduce on the number of girls you are to produce then you are reducing on your wealth”. Male Key Informant, Bugiri

In some cases, having as many children as possible was believed to be an inherent and religious directive for couples of reproductive age. Conservative points of view challenged the moral legitimacy of family planning and perceived it as undermining the husband’s fertility desires.

“If it were possible, family planning should be removed or stopped from this country and whoever talks about it should be imprisoned. Because we have been told to give birth to as many children as possible. Now if you tell me that you have brought a plan to hinder me from giving birth to many children that is not good at all”. Male FGD Participant, Mpigi

Promotion of modern contraceptives as a method for birth spacing was also viewed critically by some women who felt such messaging further led to men’s negative views regarding participation in family planning.

“The reasons as to why they are not involved is that our men love having children so much. When you reach 3–4 years without conceiving or delivering for them a baby they feel bad. Yet, as a woman you are mindful of your health”. Female FGD Participant, Bugiri

### Fear of partner sexual promiscuity

Participants also voiced concerns that women’s utilization of family planning services may lead them to become unfaithful and reflect women’s intentions to avoid pregnancy within extramarital sexual relationships. Men’s fears regarding women’s perceived sexual promiscuity was additionally linked with stigmatizing beliefs that contraception was most often used in contexts of female commercial sex exchange. It was not considered acceptable for faithful, married women. Both men and women expressed views that men’s anxiety regarding their spouse’s potential infidelity was a formidable barrier in defining supportive male roles in the utilization of reproductive health services.

“The few women who use contraceptives are seen as prostitutes. And usually men refuse their wives to associate with them because they think they will teach them how to use these contraceptives. Yet, men do not want them at all”. Male Key Informant, Bugiri

“Mostly us in the villages, someone who enters in that system of using modern contraceptives we think of her has being among the people who sell themselves or prostitutes. [In] our community, we take that person to be among those who sell themselves [as] prostitutes”. Male FGD Participant, Bugiri

“Men have a negative attitude towards contraceptives. They say they are bad, [and that]…they encourage women to move out with other men”. Female Key Informant, Bugiri

Furthermore, participants noted men’s efforts to defend against other men’s sexual interests in their spouse. Family planning methods were described as enhancing women’s physical attractiveness by delaying or preventing childbearing, which made men reluctant to support women’s contraceptive use. In exceptional cases, women who practiced family planning were also shunned by community members due to perceptions that they were intentionally abandoning the marital relationship.

“They do not want their women to stop producing because when they do, they will look healthy and younger. Men have fears that when their wives look younger, they end up attracting other men”. Female FGD Participant, Bugiri

“The men have other fears of losing their wives especially in case they use contraceptive, it makes them delay and look nice so can easily be taken by other men”. Female FGD Participant Bugiri

## Discussion

Reproductive health decision-making is the shared responsibility of men and women. Growing evidence suggests that involving men in family planning can increase women’s contraceptive uptake. Yet, in many sub-Saharan African settings, few men are involved in issues relating to reproductive health, and there is a dearth of evidence on barriers to men’s constructive engagement. This study found that many participants perceive men to be obstacles to women’s utilization of family planning, and largely uninvolved despite the fact that men are often responsible for decisions which affect the household. This was attributed to men’s reluctance to support use of modern contraceptive methods for their spouses or themselves based on fears of harmful side effects and spousal infidelity, as well as preferences for large-sized families. Institutional and social norms which define reproductive health as a “woman’s issue” and the limited choice of available male contraceptives were also cited as reasons for men’s lack of involvement. There was a common impression that such barriers hindered men’s positive and constructive participation such as discussing the couple’s fertility preferences, accompanying partners to seek reproductive health services, or providing other forms of support. Such sentiments were observed in the majority of focus groups, including male and female groups, in addition to key informant interviews.

Several important lessons emerge from the study which should be considered for future interventions. One key finding relates to comments by participants that men’s lack of involvement from fear and negative health beliefs stemmed from their overall lack of knowledge. This was attributed to the limited number of community-level reproductive health campaigns which targeted men. As a result, the emphasis on barriers related to harmful side effects may reflect heightened perceptions rather than actual experiences based on men’s reliance on informal information sources, such as other male colleagues or relatives. Our findings are consistent with current research which points to a need to better educate men in the public sphere with appropriately tailored health messaging [23-26,37].

Secondly, the finding that men were often the principal payers of health care costs relating to treatment of side effects should not be ignored. Men were aware of the direct and indirect costs of family planning services, but may not have associated such costs with long-term savings and investment. Although the role of paying for treatment of side effects was not viewed favorably by men, it highlighted their assumed financial responsibility for the family. Efforts to implement male-involvement family planning interventions should aim to encourage men’s positive role as a financial supporter and consumer of contraceptive services in ways that are mutually acceptable to men and women. This includes addressing men’s views that safe child spacing or limiting overall family size will have negative economic consequences. Both men and women also perceived discussing reproductive health matters with men as a “waste of time” since men were viewed as “too busy” generating income for the family. This highlights the economic context in which men’s participation in family planning is viewed. In contrast, although the preference for large families was a recurrent theme in our study and in previous published research [24,25], some male participants desired to have fewer children given the growing costs associated with raising a family. This suggests that despite the influence of other factors on uptake of contraceptive methods [38], economic interventions which address men’s perceptions of the direct and indirect financial costs of family planning may also be effective. This includes information on how family planning may contribute to greater financial security or interventions which integrate economic-based incentives or asset development approaches for men. Our study found that men’s interests in household financial matters, by their own report and that of women, demonstrated a need to include information on the economic implications of fertility regulation within male-focused strategies.

A third key finding was men’s interests in the development of convenient and short-term male contraceptives to replace the present-day options of male condoms and vasectomy. The perception that male condoms hinder sexual spontaneity and enjoyment is well known [39], and long-term methods such as vasectomy are not appropriate for couples who intend to conceive in the future. On the one hand, such requests reveal the unmet need of male fertility regulation methods, at least among men who are not opposed to family planning. On the other hand, these findings also demonstrate the complexity of promoting gender-equitable reproductive planning. Although men reported that family planning was a “woman’s domain,” it did not appear that women were empowered to make the final decision around contraception without significant consequences and stigma. Proposing more options for male-controlled contraceptives may reflect men’s desire to have greater influence in matters of reproductive health than is possible with female-controlled methods. Research in other settings has suggested as well that men’s opposition to family planning is a self-protective concern for themselves, rather than their partners, to mitigate suspicions of extramarital relations [39].

Lastly, the study contributes to the growing discourse on the need for gender-transformative programs in maternal and reproductive health [40]. Much of the male-involvement evidence base has examined men’s participation in the context of prevention of mother-to-child transmission and safe motherhood interventions [40], with fewer initiatives in reproductive health. Our findings showed that encouraging increased male responsibility for family planning will require careful consideration not only to counter men’s negative health beliefs around contraception, but also to shift social and institutional norms which shape how and why men are involved, including raising men’s awareness on the importance of women’s power in reproductive health decision-making. While research has shown that spousal communication and approval are significant determinants to women’s decisions to use modern contraceptives [6], it will be critical to ensure that male-focused interventions do not bypass efforts to empower women by reinforcing gender inequities, rather than challenging them [24]. Some programs have inadvertently relied on men’s unequal power to increase acceptance of health services, with little input from women [40], while women-only approaches to family planning have proven inadequate [26]. Many also have been criticized for placing undue burden on women to initiate male-involvement strategies on their own [7]. Future research should examine the comparative effectiveness of male-involvement strategies which promote gender equity by empowering women as well as increasing the positive participation of men [17,41].

## Conclusion

Joint reproductive health decision-making among couples which does not neglect the added value of men’s participation is urgently needed. The findings from this study can be used to develop effective male-involvement family planning initiatives which address barriers to men’s supportive participation in reproductive health, including addressing men’s negative health beliefs regarding contraceptive services.

## Abbreviations

FGD: Focus group discussion; KII: Key informant interview; MDG: Millennium development goal.

## Competing interests

The authors declare that they have no competing interests.

## Authors’ contributions

KA and LA conceptualized and designed the study. As principal investigator, KA was responsible for all aspects of data collection, coding, analysis, and writing of the initial manuscript draft. LJ assisted in the interpretation of findings, provided important scientific content, and wrote and revised several sections of the manuscript. AR, LA, GN, and JN critically edited drafts and added substantive intellectual content. All authors have read and approved the final version of the manuscript.

## Authors’ information

AK is a doctoral student and an assistant lecturer in the Department of Population studies, College of Business and Management Sciences, Makerere University, Kampala, Uganda. LJ is an assistant professor in the Department of International Health, Social and Behavioral Interventions Program, Johns Hopkins Bloomberg School of Public Health, Baltimore, Maryland, USA. AR is a senior researcher in the Department of Geography, University of Cambridge, United Kingdom. GN is a lecturer in the Department of Nursing, College of Health Sciences, Makerere University, Kampala, Uganda. JN is a professor in the Department if Population Studies, Makerere University, Kampala, Uganda and at the Center for Population and Statistics, College of Business and Management Sciences, Makerere University, Kampala, Uganda. LA is a lecturer in the Department of Community and Behavioral Sciences, School of Public Health, College of Health Sciences, Makerere University, Kampala, Uganda.
